# Association of lipid accumulation product trajectories with 5-year incidence of type 2 diabetes in Chinese adults: a cohort study

**DOI:** 10.1186/s12986-019-0399-7

**Published:** 2019-10-21

**Authors:** Guangyu Yan, Fei Li, Christelle Elia, Yating Zhao, Jiangguang Wang, Zhiheng Chen, Hong Yuan, Yao Lu

**Affiliations:** 1 Center of Clinical Pharmacology, The Third Xiangya Hospital, Central South University, 138 Tongzipo Road, Changsha, Hunan 410013 People’s Republic of China; 2 Department of Life Science and Medicine, King’s College London, WC2R 2LS, London, UK; 3 Health Management Center, The Third Xiangya Hospital, Central South University, 138 Tongzipo Road, Changsha, Hunan 410013 People’s Republic of China

**Keywords:** Lipid accumulation product, Trajectory, Type 2 diabetes, GLM

## Abstract

**Background:**

Lipid accumulation product (LAP) is an index describing the overaccumulation of lipid. Baseline LAP was used for type 2 diabetes (T2D) prediction in previous studies. But the longitudinal trajectories of LAP, which reflect the efficacy of patients’ lipid-lowering treatment and lifestyle improvement, have rarely been studied. The aim of this study is to explore the association of lipid accumulation product trajectories with 5-year incidence of type 2 diabetes.

**Methods:**

This cohort study included 4508 non-diabetic participants with a median age of 42 years. Using the group-based trajectory modeling (GBTM), LAP from 2011 to 2016 were determined and identified as three trajectories: low (*n* = 3639), moderate (*n* = 800), and high (*n* = 69). Baseline LAP was divided into groups by percentiles and tertiles respectively for the comparison of LAP trajectories. The associations between 5-year T2D incidence and LAP trajectories and baseline LAP were both assessed by generalized linear models.

**Results:**

From 2011 to 2016, 169 participants developed T2D (the 5-year incidence of 3.8%). For participants with low, moderate, and high trajectories, the incidence of T2D was 2.1, 10.0, and 15.9%, respectively. A significant trend was observed in the relative risks (RRs) of 5-year incident T2D in participants with moderate (RR, 1.95; 95% CI: 1.41–2.70) and high LAP trajectory (RR, 2.20; 95% CI: 1.12–4.30) in the fully adjusted model (*p* for trend< 0.001). However, there were no statically significant trends in RRs in different tertiles of baseline LAP found after full adjustments.

**Conclusion:**

The trajectories of LAP has an independent effect on 5-year T2D incidence beyond LAP measured at baseline.

## Background

Dysfunctions in adipose tissue metabolism have a direct impact on lipid and glucose homeostasis [1]. In the latest decades, a view emerged that type 2 diabetes (T2D) may reflect the complex metabolic consequences caused by the overaccumulation of ectopic lipids or hepatic fat [2–4]. And multiple epidemiological surveys have revealed that waist circumference, a routine measurement for abdominal obesity, along with hypertriglyceridemia are risk factors of T2D [5–8].

Therefore, lipid accumulation product (LAP) which calculated by waist circumference (WC) and fasting triglycerides (TG) concentration is proposed to describe lipid overaccumulation [9] and has been found higher in diabetic patients [10, 11]. Studies revealed that LAP had the potentials to predicting the risk of diseases such as cardiovascular disease (CVD) [12–14], non-alcoholic fatty liver disease [15] and metabolic syndrome [16, 17] in some healthy populations. Furthermore, LAP had been proved to be a strong predictor of diabetes occurrence in non-diabetic populations [18, 19].

Multiple cross-sectional studies [10, 11, 20], as well as some cohorts [21–23], have been conducted to investigate the associations between LAP and diabetes, but only baseline LAP was studied. The longitudinal trajectories of LAP, which may reflect the long-term effect of lipid overaccumulation, had not been reported yet, especially when concerning its association of T2D incidence. The aim of this cohort study was to explore the association of LAP trajectories with the 5-year incidence of T2D in a Chinese population.

## Methods

### Study population

**Fig. 1:**
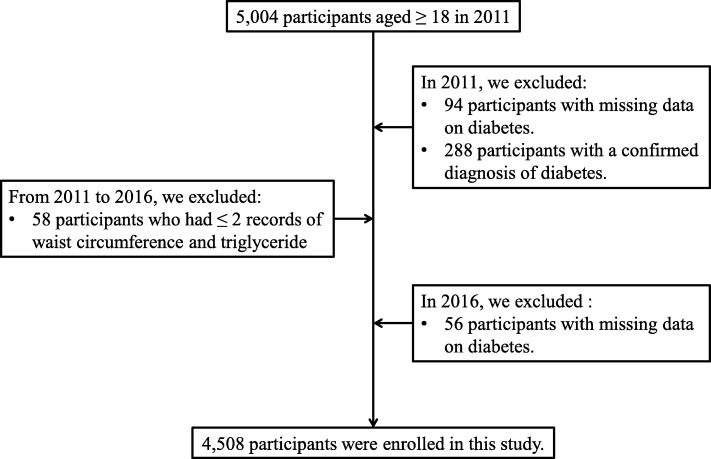
A flow diagram of participant screening and enrollment

In this study, we retrospectively recruited 5004 participants over 18 years old from the institutions and had completed a medical examination annually from 2011 to 2016 in the Health Management Center of Third Xiangya Hospital, which is one of the largest medical examination centers in China. The exclusion criteria (Fig. 1) were as follows: 1) participants with missing data on the diagnosis of diabetes or confirmed diagnosis of diabetes in 2011, 2) participants with missing data on the diagnosis of diabetes in 2016, 3) participants with less than two records of both WC and TG.

The present study was in accordance with the guidelines of the Declaration of Helsinki and was approved by the Medical Ethics Committee of Third Xiangya Hospital. All the participants have signed an informed consent form and agreed to share their health information for medical research.

### Diagnosis of type 2 diabetes

Blood samples from all participants were collected in the morning after overnight fasting for 8–12 h and then stored at EDTA-containing vacuum tubes. All blood samples were stored in freezers at − 20 °C until analyzed. The concentration of fasting blood glucose (FBG) was measured by enzymatic colorimetric assay using an automated analyzer (Hitachi 7600–110; Hitachi, Tokyo, Japan) at the central laboratory of the Third Xiangya Hospital. T2D was defined by the presence of any of the following: 1) self-reported doctor-diagnosed type 2 diabetes, 2) current use of insulin or oral hypoglycemic agents, and 3) fasting blood glucose ≥7.0 mmol/L [24].

### Assessment of LAP

WC was measured at the navel level using an un-stretched tape measure and without applying any pressure to the body surface [15]. The concentration of fasting TG was measured from the same blood samples and by the same automated analyzer mentioned above. LAP was calculated using the formula [WC (cm) – 65] × TG concentration (mmol/L) for men, and [WC (cm) – 58] × TG concentration (mmol/L) for women [9].

### Assessment of other potential covariates

All demographic data (age, sex, race, and marital status), lifestyle factors (cigarette smoking and alcohol consumption), and medical history were obtained by well-trained interviewers using standardized questionnaires. Eleven racial groups, including Han, Miao, Tujia, Hui, Zhuang, Bai, Dong, Man, Yao, Mongolian, and Xibo, were identified in our study and divided into two categories: Han and all other minorities. Marital status was defined as married and unmarried. And all widowed or divorced subjects were defined as married.

Bodyweight and height were measured to the nearest 0.1 kg or 0.1 cm, with participants wearing light clothes and no shoes. Body mass index (BMI) was calculated as body weight in kilograms (kg) divided by the square of height in meters (m^2^). Blood pressure (BP) was measured from participants in a sitting position after a 10-min rest. Using a corrected mercury sphygmomanometer, two readings were obtained with an interval of 30 s for both systolic and diastolic BP. The mean of the two readings was considered as the participant’s BP. If the two readings differed by > 5 mmHg, BP was re-measured, and the participant’s BP was finally calculated as the average of the three readings. Hypertension was defined as 1) systolic BP ≥140 mmHg and/or diastolic BP ≥ 90 mmHg, 2) self-reported or doctor-diagnosed hypertension, 3) current use of antihypertensive agents [25].

All the other biochemical measurements, including total cholesterol, hemoglobin, creatinine, and uric acid, were measured from the same blood samples and by the same automated analyzer mentioned above [15, 25].

### Statistical analysis

All statistical analyses were conducted using Stata version 14.0 (Stata Corp., College Station, TX). The LAP trajectories were formed by group-based trajectory modeling (GBTM), which we also fitted in conjunction with the user-written “traj” program in Stata [26, 27]. GBTM is considered to be a potential growth curve analysis that identifies individuals with a similar trajectory that tracks a single indicator [26]. LAP was modeled by a censored normal distribution. The model with the best fitness was chosen by the following steps [28]: 1) choose an optimal number of trajectories from models with the same shapes but different numbers of trajectories. 2) adjust the shape orders of the model with the optimal number of trajectories and then choose the best-fit model, 3) draw a plot of trajectories from the best-fit model and identify the trajectories. The number and shapes of the LAP trajectories (i.e., linear, quadratic, and cubic) were chosen by following criteria: a) highest value of Bayesian Information Criterion, b) high posterior probability (> 0.7), c) enough membership in allocated groups (at least 1%). A plot of trajectories will be drawn for the best-fit model, and trajectories will be identified according to their different levels.

The baseline characteristics of this study were summarized as the median (interquartile range) for continuous variables and the number (percentage) for categorical variables. The Kruskal-Wallis test for continuous variables and the chi-square test for categorical variables were used to compare to the baseline characteristics of the patients with different LAP trajectories.

Missing values of potential covariables that occurred during the investigation were processed by multiple imputations with chained equations [29]. Thirty imputed datasets were generated for the imputed models since approximately 30% of the study samples had missing values in at least one covariable [29]. And all variables of this study were added into the imputation models in order to achieve better imputation [29].

To compare with the LAP trajectories, baseline LAP was divided into two methods: tertiles and percentiles. Percentiles of baseline LAP was decided by the proportions of LAP trajectories (e.g., if the proportion of LAP trajectories was 20, 40, and 40%, the percentiles groups should be P_0_ - P_20_, P_20_ - P_60_, and P_60_ - P_100_). Baseline LAP and LAP trajectories were analyzed simultaneously to determine the associations with 5-year T2D. The relative risk (RR) of new-onset diabetes in 2016 was used as the parameter of interest to correct for the overestimation in estimates of effect observed with odds ratios when the outcome rate exceeds 10% [30]. Therefore, generalized linear models (GLM) with log-link function, Poisson distributions, and robust error variance were used in this study for yielding RR estimates [31]. The goodness-of-fit was measured as averaged Akaike information criterion (AIC) of GLM models in 30 imputed datasets [32], and analysis of variance (ANOVA) was used to examine the differences among GLM models.

## Results

### Baseline characteristics by three trajectories of LAP

Totally 4508 participants (54.1% males) were enrolled in the study with a median age of 42.0 years old. The baseline characteristics of the participants are presented in Table 1. The mean BMI and LAP in all participants was 23.4 kg/m^2^ and 19.7 respectively. And the average fasting blood concentrations of glucose, total cholesterol (TC) triglycerides (TG) and high-density lipoprotein cholesterol (HDL) was 5.0, 4.7, 1.14 and 1.40 mmol/L respectively (Table 1).

**Table 1:** Baseline characteristics according to LAP trajectories

Variables^a^	Total (*n* = 4508)	Low trajectory (*n* = 3639)	Moderate trajectory (*n* = 800)	High trajectory (*n* = 69)	*P* value^b^
Age (years)	42.0 (32.0–57.0)	40.0 (31.0–57.0)	47.0 (38.0–60.8)	40.0 (33.0–48.0)	**< 0.001**
Male (%)	2438 (54.1)	1723 (47.4)	648 (81.0)	67 (97.1)	**< 0.001**
BMI (kg/m^2^)	23.4 (20.9–25.6)	22.5 (20.5–24.7)	26.3 (24.6–28.0)	27.3 (25.4–29.2)	**< 0.001**
WC (cm)	80.0 (71.0–87.0)	76.0 (70.0–84.0)	89.0 (85.0–94.0)	92.0 (88.0–95.8)	**< 0.001**
Han ethnicity (%)	4339 (96.6)	3500 (96.5)	773 (97.2)	66 (95.7)	0.513
Current smoker (%)	990 (26.6)	614 (20.5)	331 (49.9)	45 (75.0)	**< 0.001**
Current alcohol drinker (%)	2031 (60.2)	1490 (55.0)	487 (80.0)	54 (90.0)	**< 0.001**
Married status (%)	3412 (86.4)	2677 (84.5)	680 (94.2)	55 (93.2)	**< 0.001**
Family history of diabetes (%)	347 (7.7)	279 (7.7)	59 (7.4)	9 (13.0)	0.235
Hypertension (%)	947 (21.7)	634 (18.0)	295 (37.7)	18 (27.3)	**< 0.001**
Use of antihypertensive (%)	407 (9.0)	285 (7.8)	118 (14.8)	4 (5.8)	**< 0.001**
LAP	19.7 (9.9–36.0)	15.4 (8.0–25.7)	56.6 (43.0–75.8)	118.5 (86.6–200.1)	**< 0.001**
Fasting blood concentrations of					
Glucose (mmol/l)	5.0 (4.7–5.3)	4.9 (4.7–5.3)	5.2 (4.8–5.6)	5.2 (4.8–5.8)	**< 0.001**
Total cholesterol (mmol/l)	4.7 (4.2–5.3)	4.6 (4.1–5.2)	5.1 (4.5–5.8)	5.7 (4.9–6.3)	**< 0.001**
Triglycerides (mmol/l)	1.14 (0.79–1.67)	1.00 (0.73–1.35)	2.28 (1.71–2.98)	4.5 (3.2–8.1)	**< 0.001**
HDL (mmol/l)	1.40 (1.15–1.71)	1.48 (1.23–1.78)	1.14 (0.96–1.32)	1.04 (0.85–1.21)	**< 0.001**
Hemoglobin (g/L)	133.0 (124.0–144.0)	131.0 (123.0–142.0)	144.0 (135.0–150.0)	146.0 (138.0–154.0)	**< 0.001**
Creatinine (μmol/L)	66.0 (54.0–78.0)	62.0 (53.0–75.0)	75.0 (66.0–84.0)	74.0 (65.0–83.0)	**< 0.001**
Uric acid (μmol/L)	283.0 (221.0–350.0)	263.0 (210.0–327.0)	359.0 (305.0–418.0)	390.0 (332.0–451.5)	**< 0.001**

*BMI* body mass index, *WC* waist circumference, *LAP* lipid accumulation product, *HDL* high-density lipoprotein cholesterol

^a^Continuous variables were summarized as median (interquartile range)

^b^*P* values are calculated by Kruskal–Wallis test for continuous variables and chi-square test for categorical variables

**Fig. 2:**
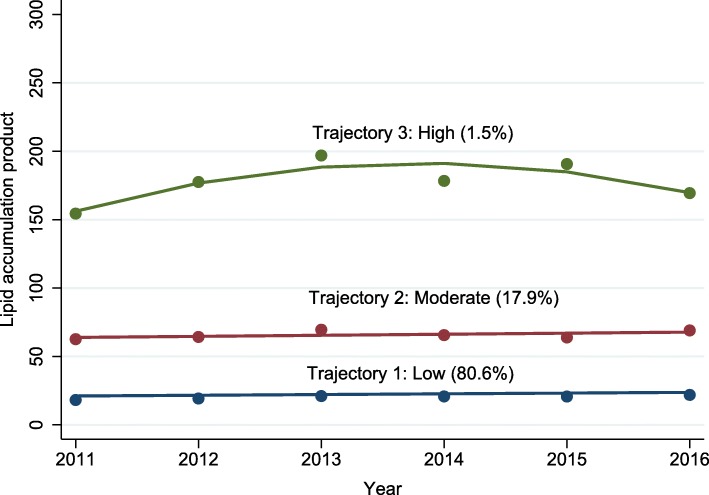
Three LAP trajectories determined by the best-fit model from 2011 to 2016

After the examination of all fitting results (Additional file 1: Tables S1 and S2), a best-fit model with three trajectories is determined and identified as low, moderate, and high. From 2011 to 2016, the low and moderate LAP trajectory presented a slight elevation, while the high LAP trajectory rose at first and then declined (Fig. 2). Models with 2 or 4 trajectories were omitted since they were either poor in Bayesian Information Criterion or lacked subjects in the trajectory group.

Participants in low trajectory group (80.7% of participants were in this group), compared to those in moderate (17.8%) or high trajectory group (1.5%), were less likely to be male, had lower BMI at the baseline, and were not prone to be current smoker nor alcohol drinker nor hypertensive. Higher FBG, TC, TG, creatinine and uric acid and lower HDL were found in moderate or high trajectory group, compared with low trajectory group.

### Association between the 5-year incidence of T2D and LAP trajectories

From 2011 to 2016, 169 participants developed T2D, and the overall incidence of T2D was 3.8% (Table 2). The incidence of T2D progressively increased at higher levels of LAP trajectories. For participants with low, moderate and high trajectories, the incidence of T2D was 2.1, 10.0, and 15.9%, respectively (Table 2).

**Table 2:** Relative risk and 95% confidence interval for the 5-year incidence of type 2 diabetes according to LAP trajectories and baseline LAP

Variables	*N*	Cases (%)	Model 1^a^ Adjusted RR (95% CI)	Model 2^b^ Adjusted RR (95% CI)	Model 3^c^ Adjusted RR (95% CI)
LAP trajectories	4508	169 (3.8)			
Trajectory 1: low	3639	78 (2.1)	1.00 (Ref)	1.00 (Ref)	1.00 (Ref)
Trajectory 2: moderate	800	80 (10.0)	3.51 (2.57–4.80)	2.56 (1.78–3.70)	1.95 (1.41–2.70)
Trajectory 3: high	69	11 (15.9)	7.68 (4.12–14.32)	5.20 (2.77–9.76)	2.20 (1.12–4.30)
*P* value for trend			**< 0.001**	**< 0.001**	**< 0.001**
Baseline LAP (groups)^d^	4179	154 (3.7)			
First group: (< 42.4)	3368	69 (2.1)	1.00 (Ref)	1.00 (Ref)	1.00 (Ref)
Second group: (42.4 ≤ ~ < 121.6)	748	78 (10.4)	3.60 (2.61–4.96)	2.56 (1.76–3.73)	1.73 (1.25–2.38)
Third group: (≥121.6)	63	7 (11.1)	4.62 (2.21–9.66)	2.61 (1.15–5.95)	1.47 (0.66–3.31)
*P* value for trend			**< 0.001**	**< 0.001**	0.006
Baseline LAP (tertiles)	4179	154 (3.7)			
First tertile: (< 12.7)	1393	12 (0.9)	1.00 (Ref)	1.00 (Ref)	1.00 (Ref)
Second tertile: (12.7 ≤ ~ < 29.3)	1394	27 (1.9)	1.33 (0.67–2.66)	1.04 (0.52–2.07)	1.03 (0.52–2.03)
Third tertile: (≥29.3)	1392	115 (8.3)	4.90 (2.65–9.06)	2.93 (1.52–5.65)	1.91 (0.97–3.74)
*P* value for trend			**< 0.001**	**< 0.001**	0.007

*LAP* lipid accumulation product

^a^Model 1: adjusted for baseline age, gender, race, current smoking, current alcohol drinking, and married status

^b^Model 2: further adjusted for baseline diagnosis of hypertension, use of antihypertensive, total cholesterol, high-density lipoprotein cholesterol, hemoglobin, creatinine, and uric acid

^c^Model 3: further adjusted for baseline blood glucose and family history of diabetes

^d^From first to the third group, the proportions of subjects were 80.6, 17.9, and 1.5%, respectively. The divisions were consistent with the proportions of different LAP trajectories

Compared with the low trajectory, significant trends were observed in RRs of 5-year incident T2D in participants with moderate (RR, 2.56; 95% confidence interval [CI]: 1.78–3.70) and high LAP trajectories (RR, 5.20; 95% CI: 2.77–9.76), respectively (*p* for trend < 0.001) in GLM adjusted for sociodemographic variables, behavioral factors, hypertension, lipid profile, hemoglobin, creatinine and uric acid (Table 2, Model 2). These associations remained significant after further adjustment for baseline FBG and family histories of diabetes. The RR for moderate trajectory was 1.95 (95% CI: 1.41–2.70) and for high trajectory was 2.20 (95% CI: 1.12–4.30) compared with the low trajectory (*p* for trend < 0.001) (Table 2, Model 3). The goodness-of-fit was improved from model 1 to model 3 (Additional file 1: Table S3).

### Association between the 5-year incidence of T2D and baseline LAP

GLM was performed in groups determined by percentiles (P_80.6_ and P_98.5_) and tertiles according to baseline LAP. After adjusted for sociodemographic variables, behavioral factors, hypertension, lipid profile, hemoglobin, creatinine and uric acid (Table 2, Model 2), a significant trend (*p* for trend < 0.001) in relative risks for 5-year incident T2D over follow-up was observed in second group (RR, 2.56; 95% CI: 1.76–3.73) and third group (RR, 2.61; 95% CI: 1.15–5.95), compared with the first group respectively. And a significant trend (*p* for trend < 0.001) in relative risks was also found in second tertile (RR, 1.04; 95% CI: 0.52–2.07) and third tertile (RR, 2.93; 95% CI: 1.52–5.65), compared with the first tertile group (Table 2, Model2). However, significant associations between baseline LAP and 5-year incident T2D disappeared after further adjustment for baseline blood glucose and family history of diabetes (p for trend was 0.006 and 0.007 respectively) (Table 2, Model3). The goodness-of-fit was also improved from model 1 to model 3 (Additional file 1: Table S3).

## Discussion

In the current cohort study, we found that higher LAP trajectories were associated with an increased risk of diabetes. Compared to baseline LAP, trajectories of longitudinal LAP had a stronger relationship with incident T2D, especially after adjusting for more confounders.

Overaccumulation of ectopic lipids or hepatic fat causes serials of complex metabolic consequences [2–4] such as non-alcoholic fatty liver disease and insulin resistance, which will finally promote the development of diabetes. Recent studies have focused on diabetic correlations in longitudinal changes in multiple indices reflecting obesity and lipid metabolism, such as BMI [33, 34], visceral adiposity index [35], and lipids [36]. LAP combines waist measurements and fasting triglyceride (TG) levels, reflecting both the anatomic and physiological changes associated with lipid overaccumulation. LAP has been confirmed to be closely associated with diabetes, metabolic syndrome, and cardiovascular diseases, and outperformed BMI for identifying these diseases [9, 11, 18, 19]. Accordingly, it is plausible that the longitudinal change of LAP is significantly associated with T2D.

The association between baseline LAP and the incidence of diabetes has been investigated in several different ethnic cohorts. A cohort study of 4083 Chinese subjects indicated that higher LAP was associated with increased RR of diabetes (*p* for trend < 0.05 from Q1 to Q4 in both male and female) [22]. A Korean cohort study reported that the adjusted ORs for the highest quartiles of LAP, compared to the lowest, was 2.47 (95% CI: 1.82–3.34) in men and 2.44 (95% CI: 1.82–3.26) in women respectively [22]. The result in a 6-year follow-up study of Tehran suggested that OR for incident diabetes with 1 standard deviation increment (log-scale) of LAP was 2.3 (95% CI: 1.8–3.0) for males and 3.2 (95% CI: 2.6–4.1) for females at the age of 20–49 [19]. Similar results had also been reported in Korea [20], Montenegro [37], Italy [38], Germany [21], and the United States [9].

Though the association between baseline LAP and diabetic incidence has been studied, little was known about how the longitudinal effect of LAP trajectories on T2D incidence. The longitudinal level of LAP, as well as the risk of diabetes, are both influenced by many factors. In our current study, moderate (81.0%) and high (97.1%) LAP trajectory consisted of a great majority of males with a higher incidence of diabetes. Similar to this result, Wakabayashi et al. reported that prevalence of high LAP was higher in males than in females before the age of 50 (males vs. females, 34.4% vs. 40.8% at age of 35–39 years; 13.1% vs. 18.1% at age of 40–49 years), but this difference was narrowed by aging, even leading a reversed result in the age group of 60–70 years (38.4% vs. 35.6%) [39]. Besides, elevated LAP in postmenopausal women with diabetes, considering the decline of estrogen, would lead to fat redistribution [21]. Therefore, only baseline LAP may not be sufficient to reflect changes in the longitudinal LAP and its relationship to incident diabetes. In our study, the association between LAP trajectories and T2D incidence was firstly reported that participants with moderate and high LAP trajectories were at higher risks of T2D, compared to those in low trajectories. Our study indicated that compared with higher baseline LAP, higher longitudinal trajectories of LAP were more closely associated with T2D incidence, which implied the insufficiency in focusing baseline LAP alone.

The strengths of our study included a longitudinal design, large-sample size with repeated LA*P* values over time and the use of GBTM. As a novel index, LAP is radiation-free, simple, cheap, and easy to perform, compared with CT (computed tomography) and MRI (magnetic resonance imaging) for body fat measurement [18]. However, this study has some limitations. Firstly, lacking the data of using oral glucose tolerance tests to diagnose diabetes may lead to underestimation of diabetes events. Secondly, due to the relatively small sample size, further analyses with stratification by gender (only two females in high trajectory group) couldn’t be conducted.

## Conclusion

Our study identified three distinct trajectories (low, moderate, and high) of LAP and the trajectories of LAP has an independent effect on T2D incidence beyond LAP measured at baseline. The results suggest that the longitudinal trajectories of LAP may be an important risk factor of diabetes.

## Supplementary information

**Additional file 1: Table S1.** Model fit statistics by the numbers of trajectories. **Table S2.** Model fit statistics of 27 combinations of shape orders (model with 3 trajectories). **Table S3.** AIC^a^ for model 1 to model 3 performed in 30 datasets generated from multiple imputation. **Table S4.** Summary of baseline LAP by LAP trajectory groups, percentiles groups of baseline LAP, and tertiles of baseline LAP.

## Data Availability

The datasets analyzed during the current study are available from the corresponding author on reasonable request.

## Abbreviations

AIC: Akaike information criterion
ANOVA: Analysis of variance
BMI: Body mass index
BP: Blood pressure
CI: Confidence interval
CT: Computed tomography
CVD: Cardiovascular disease
FBG: Fasting blood glucose
GBTM: Group-based trajectory modeling
GLM: Generalized linear model
HDL: High density lipoprotein
HR: Hazard ratio
LAP: Lipid accumulation product
MRI: Magnetic resonance imaging
OR: Odds ratio
RR: Relative risk
T2D: Type 2 diabetes
TC: Total cholesterol
TG: Triglycerides
WC: Waist circumference
