# Supplementary material for: Association of lipid accumulation product trajectories with 5-year incidence of type 2 diabetes in Chinese adults: a cohort study
Source: Nutr Metab (Lond). 2019 Oct 21;16:72. doi: 10.1186/s12986-019-0399-7 (PMC6802349; doi:10.1186/s12986-019-0399-7)
Supplement: Supplementary file 1 — Additional file 1: Table S1. Model fit statistics by the numbers of trajectories. Table S2. Model fit statistics of 27 combinations of shape orders (model with 3 trajectories). Table S3. AICa for model 1 to model 3 performed in 30 datasets generated from multiple imputation. Table S4. Summary of baseline LAP by LAP trajectory groups, percentiles groups of baseline LAP, and tertiles of baseline LAP. [file 12986_2019_399_MOESM1_ESM.docx]

**Table S1.** Model fit statistics by the numbers of trajectories.

| Number of trajectories | Parameter of trajectory shape ^a^ | Allocated group membership | Estimated group membership | AvPP ^b^ | BIC ^c^ | AIC ^c^ |
| --- | --- | --- | --- | --- | --- | --- |
| 2 | 2  2 | 91.32%  8.67% | 91.24%  8.76% | 0.99  0.97 | -118236.52 | -118210.86 |
| **3** | **2**  **2**  **2** | **18.05%**  **80.28%**  **1.66%** | **18.18%**  **80.16%**  **1.66%** | **0.94**  **0.99**  **0.99** | **-115242.83** | **-115204.35** |
| 4 | 2  2  2  2 | 3.68%  73.67%  22.14%  0.51% | 3.71%  73.48%  22.30%  0.51% | 0.98  0.98  0.93  0.99 | -113967.91 | -113916.61 |

AvPP, average posterior possibility; BIC, Bayesian information criterion; AIC, Akaike information criterion.

^a^ Polynomial function of time (1 linear, 2 quadratic, 3 cubic).

^b^ AvPP >0.7 indicates good classification accuracy.

^c^ Lower absolute values of BIC and AIC indicates better fitness.

**Table S2.** Model fit statistics of 27 combinations of shape orders (model with 3 trajectories).

| Parameter of trajectory shape ^a^ | Allocated group membership | Estimated group membership | AvPP ^b^ | BIC ^c^ | AIC ^c^ |
| --- | --- | --- | --- | --- | --- |
| 1  1  1 | 80.32%  18.08%  1.60% | 80.26%  18.11%  1.62% | 0.99  0.94  0.99 | -115237.42 | -115208.56 |
| 2  2  2 | 18.05%  80.28%  1.66% | 18.18%  80.16%  1.66% | 0.94  0.99  0.99 | -115242.83 | -115204.35 |
| 3  3  3 | 37.53%  49.58%  12.89% | 33.33%  33.33%  33.33% | 0.49  0.50  0.89 | -120450.93 | -120402.83 |
| **1**  **1**  **3** | **80.72%**  **17.75%**  **1.53%** | **80.56%**  **17.91%**  **1.53%** | **0.99**  **0.94**  **0.99** | **-115195.32** | **-115160.04** |
| 1  1  2 | 18.06%  80.28%  1.66% | 18.18%  80.16%  1.66% | 0.94  0.99  0.99 | -115234.40 | -115202.33 |
| 1  2  1 | 80.32%  18.08%  1.60% | 80.28%  18.10%  1.62% | 0.99  0.94  0.99 | -115242.70 | -115210.63 |
| 2  1  1 | 80.32%  18.08%  1.60% | 80.27%  18.11%  1.62% | 0.99  0.94  0.99 | -115241.83 | -115209.76 |
| 2  2  1 | 18.08%  80.32%  1.60% | 18.10%  80.28%  1.62% | 0.94  0.99  0.99 | -115247.14 | -115211.86 |
| 2  1  2 | 18.06%  80.28%  1.66% | 18.18%  80.16%  1.66% | 0.94  0.99  0.99 | -115238.61 | -115203.34 |
| 1  2  2 | 80.28%  18.06%  1.66% | 80.16%  18.18%  1.66% | 0.99  0.94  0.99 | -115238.61 | -115203.34 |
| 1  3  1 | 18.06%  80.35%  1.60% | 18.10%  80.28%  1.62% | 0.94  0.99  0.99 | -115244.05 | -115208.77 |
| 3  1  1 | 18.06%  80.32%  1.62% | 18.14%  80.22%  1.64% | 0.94  0.99  0.99 | -115244.48 | -115209.20 |
| 1  3  3 | 80.70%  17.77%  1.53% | 80.55%  17.92%  1.53% | 0.99  0.94  0.99 | -115202.66 | -115160.97 |
| 3  3  1 | 40.91%  37.18%  21.92% | 33.33%  33.33%  33.33% | 0.48  0.49  0.84 | -120761.48 | -120719.79 |
| 3  1  3 | 53.17%  37.40%  9.43% | 33.33%  33.33%  33.33% | 0.67  0.68  0.93 | -120445.82 | -120404.13 |
| 2  2  3 | 17.72%  80.75%  1.53% | 17.91%  80.56%  1.52% | 0.95  0.99  0.99 | -115203.74 | -115162.06 |
| 3  2  2 | 80.32%  18.01%  1.66% | 80.17%  18.16%  1.66% | 0.99  0.94  0.99 | -115245.15 | -115203.46 |
| 2  3  2 | 18.01%  80.32%  1.66% | 18.16%  80.17%  1.66% | 0.94  0.99  0.99 | -115245.15 | -115203.46 |
| 2  3  3 | 50.69%  37.27%  12.05% | 33.33%  33.33%  33.33% | 0.57  0.57  0.91 | -120447.53 | -120402.63 |
| 3  2  3 | 51.02%  37.33%  11.65% | 33.33%  33.33%  33.33% | 0.58  0.59  0.91 | -120447.89 | -120402.99 |
| 3  3  2 | 44.43%  33.92%  21.65% | 33.33%  33.33%  33.33% | 0.49  0.49  0.54 | -120729.66 | -120684.76 |
| 1  2  3 | 80.75%  17.72%  1.53% | 80.56%  17.91%  1.53% | 0.99  0.95  0.99 | -115199.53 | -115161.05 |
| 1  3  2 | 80.28%  18.06%  1.66% | 80.11%  18.21%  1.68% | 0.99  0.94  0.99 | -115240.44 | -115201.96 |
| 2  3  1 | 18.01%  80.39%  1.60% | 18.09%  80.29%  1.62% | 0.94  0.99  0.99 | -115249.33 | -115210.85 |
| 2  1  3 | 80.72%  17.75%  1.53% | 80.56%  17.91%  1.53% | 0.99  0.94  0.99 | -115199.53 | -115161.05 |
| 3  1  2 | 80.32%  18.01%  1.66% | 80.17%  18.16%  1.66% | 0.99  0.94  0.99 | -115240.94 | -115202.45 |
| 3  2  1 | 18.08%  80.32%  1.60% | 18.14%  80.23%  1.63% | 0.94  0.99  0.99 | -115248.99 | -115210.51 |

AvPP, average posterior possibility; BIC, Bayesian information criterion; AIC, Akaike information criterion.

^a^ Polynomial function of time (1 linear, 2 quadratic, 3 cubic).

^b^ AvPP >0.7 indicate good classification accuracy.

^c^ Lower absolute values of BIC and AIC indicate better fitness.

**Table S3.** AIC^a^ for model 1 to model 3 performed in 30 datasets generated from multiple imputation.

|  | **Model 1** | **Model 2** | **Model 3** | ***P* value^b^** |
| --- | --- | --- | --- | --- |
| **LAP trajectories** | 0.281 (0.280, 0.282) | 0.279 (0.278, 0.280) | 0.233 (0.232, 0.234) | <0.001 |
| **Baseline LAP (groups)** | 0.278 (0.276, 0.279) | 0.276 (0.275, 0.277) | 0.229 (0.228, 0.230) | <0.001 |
| **Baseline LAP (tertiles)** | 0.277 (0.276, 0.278) | 0.275 (0.274, 0.276) | 0.229 (0.227, 0.230) | <0.001 |

AIC, Akaike information criterion; LAP, lipid accumulation product.

a AIC were calculated by each 30 datasets performed in model 1, model 2, and model 3, and are demonstrated as median (min, max). The lower AIC indicates the better fitness.

b P value from analysis of variance of AIC in model 1, model 2, and model 3.

**Table S4.** Summary of baseline LAP by LAP trajectory groups, percentiles groups of baseline LAP, and tertiles of baseline LAP.

|  | **Mean ± SD** | **Maximum value** | **Minimum value** |
| --- | --- | --- | --- |
| **LAP trajectories** |  |  |  |
| Trajectory 1: low | 18.18 ± 12.85 | 0.43 | 125.00 |
| Trajectory 2: moderate | 63.02 ± 32.67 | 5.90 | 322.16 |
| Trajectory 3: high | 154.57 ± 102.12 | 21.00 | 429.44 |
| **Baseline LAP (groups)** |  |  |  |
| First group | 17.31 ± 10.86 | 0.43 | 42.39 |
| Second group | 63.88 ± 18.50 | 42.42 | 121.40 |
| Third group | 200.23 ± 83.13 | 121.60 | 429.44 |
| **Baseline LAP (tertiles)** |  |  |  |
| First tertile | 7.06 ± 3.25 | 0.43 | 12.65 |
| Second tertile | 20.14 ± 4.83 | 12.70 | 29.38 |
| Third tertile | 58.07 ± 40.63 | 29.40 | 429.44 |
